# Structural Manipulation of Eicosanoid Receptors and Cellular Signaling

**DOI:** 10.1100/tsw.2007.163

**Published:** 2007-09-01

**Authors:** Charles Brink

**Affiliations:** INSERM U698, Haemostasis, Bio-Engineering and Cardiovascular Remodelling, CHU Xavier Bichat Secteur Claude Bernard, 46 rue Henri Huchard, 75877 Paris Cedex 18, France

**Keywords:** prostanoid receptors, leukotriene receptors, prostaglandins, leukotrienes, GPCRs, receptor structure, receptor signaling

## Abstract

Eicosanoids are lipid mediators derived from the metabolism of arachidonic acid. These agents are locally released and activate different cell membrane receptors, and the latter are part of the G-protein coupled receptor family. While activation of eicosanoid receptors is associated with a wide variety of actions, there is limited information concerning the structural components of the eicosanoid receptors. To date, our understanding of the eicosanoid ligand-receptor binding interaction has been based on the rhodopsin template model. While receptors in the same family do share a common architecture, there are amino acid residues in the membrane binding pocket that play a key role in ligand recognition as well as the diversity observed in the cellular signaling. In order to understand the eicosanoid receptor binding interaction, attention must be focused on both the nature of the endogenous ligands as well as the template G-protein model that has been proposed. The data derived from chemical alterations in the endogenous ligands, together with the mutagenesis studies involving the structural modifications of the eicosanoid receptors, have suggested a working model of the eicosanoid receptors. However, the data also document various nuances in the receptor structure associated with ligand binding as well as a number of differences that will require further investigation.

In spite of the progress that has been made in the classification of the eicosanoid receptors, our understanding of both their structures as well as their cellular signaling pathways is far from satisfactory. In general, when confronted with the diverse activities of G-protein coupled receptors (GPCRs), one is reminded of the Chinese aphorism: "*There are things which could never be imagined, but there is nothing which may not happen*".

Prostanoids have been reported to bind to a number of structurally diverse molecules, such as cell surface receptors (EP, FP, IP, DP, and TP[1]), nuclear receptors[2,3], the prostaglandin transporter[4,5] several PPAR nuclear receptors[6], and prostaglandin-15-dehydrogenase[7,8]. Similarly, leukotrienes also bind to specific membrane cell surface receptors[9], nuclear membrane PPAR receptors[10], ATP transporters[11], and, depending on the leukotriene, different cellular enzymes[12,13,14]. These observations suggest that eicosanoids are controlled by both extracellular processes as well as intracellular mechanisms.

In addition, ligand-receptor interactions are remarkably controlled at the cell membrane surface receptor. At this level, GPCR responsiveness is intimately linked and regulated by (a) *receptor density* (increased or decreased expression); (b) *phosphorylation,* which leads to uncoupling, thereby reducing the receptor-protein interactions; and (c) *sequestration/internalization* in which the receptor is either lost (degraded) or recycled. However, ligand-GPCR interactions are also dependent on the kinetic state of the receptor structure. Receptors are known to exhibit several conformations, some of which produce measurable pharmacological effects (*active* vs. *inactive states*). Since receptors may exist in an active state (constitutive activity), the ligand may not always be necessary for activation, but may simply allow this constitutive activity to persist by stabilizing the receptor in an active conformational condition.

To these general features of GPCRs must be added the notion that cells have numerous cellular mechanisms for fine tuning ligand-receptor interactions. A considerable amount of information has accrued to suggest that GPCRs interact not only with their ligands, but also with a variety of accessory cellular proteins that are responsible for GPCR targeting toward specific cellular signals. These proteins permit the GPCR membrane structure to assemble into larger functional complexes that play a major role in the regulation of the GPCR-ligand interaction. The responsiveness of the receptors at the level of the cellular membrane surface, therefore, is dependent not only on the input of information from the endogenous ligand, but also on the cellular signals and effectors associated with the GPCR. This complex structural ensemble contributes to the overall action of the ligand-receptor activation.

Most of the investigations concerning the structural modeling of the GPCRs have been based on rhodopsin as a template. Admittedly, receptors do share a common architecture, however, they have the ability to recognize and transduce structurally diverse messages to a variety of intracellular mechanisms, suggesting that there are highly specific refinements in the receptor entity that facilitate the selective actions of a diverse number of endogenous ligands. This review will address some of the general structural features of the eicosanoid receptors, and highlight those similarities and differences that have been reported.

## EICOSANOID LIGAND MODIFICATIONS

In an attempt to understand cell membrane surface receptors, information concerning the structure of the native endogenous ligand needs to be considered. This is not only helpful to understand how the receptor works, but is also a prerequisite for the development of a cell membrane receptor model. When considering the structure of the natural endogenous prostaglandins, there are at least two basic components to recall: (1) the cyclopentane ring and (2) the side chains. On the cyclopentane ring, different functional moieties are attached at C-9 and C-11, separating the prostaglandins into different groups (E, F, D, and I). In addition, the number of double bonds in the side chains permitted a further classification into different types (1, 2, and 3). The receptors, at which the prostaglandins act, therefore, must recognize all of these ligand structural characteristics. Once the structure of the native ligand was known, the molecule was modified in an attempt to characterize and identify the specific receptor at which the endogenous ligand works.

One of the most studied metabolites of the arachidonic acid enzymatic cascade is prostaglandin $E_2$. The structures of native prostanoids are presented in Fig. 1 and the principal moieties of the ligand, which are known to alter potency and affinity, are highlighted in Fig. 2. Since $PGE_2$ is known to activate a number of prostanoid receptors ($EP_1$, $EP_2$, $EP_3$, and $EP_4$), modifications in the native structure were explored in order to facilitate the development of compounds that were selective for each of these receptors.

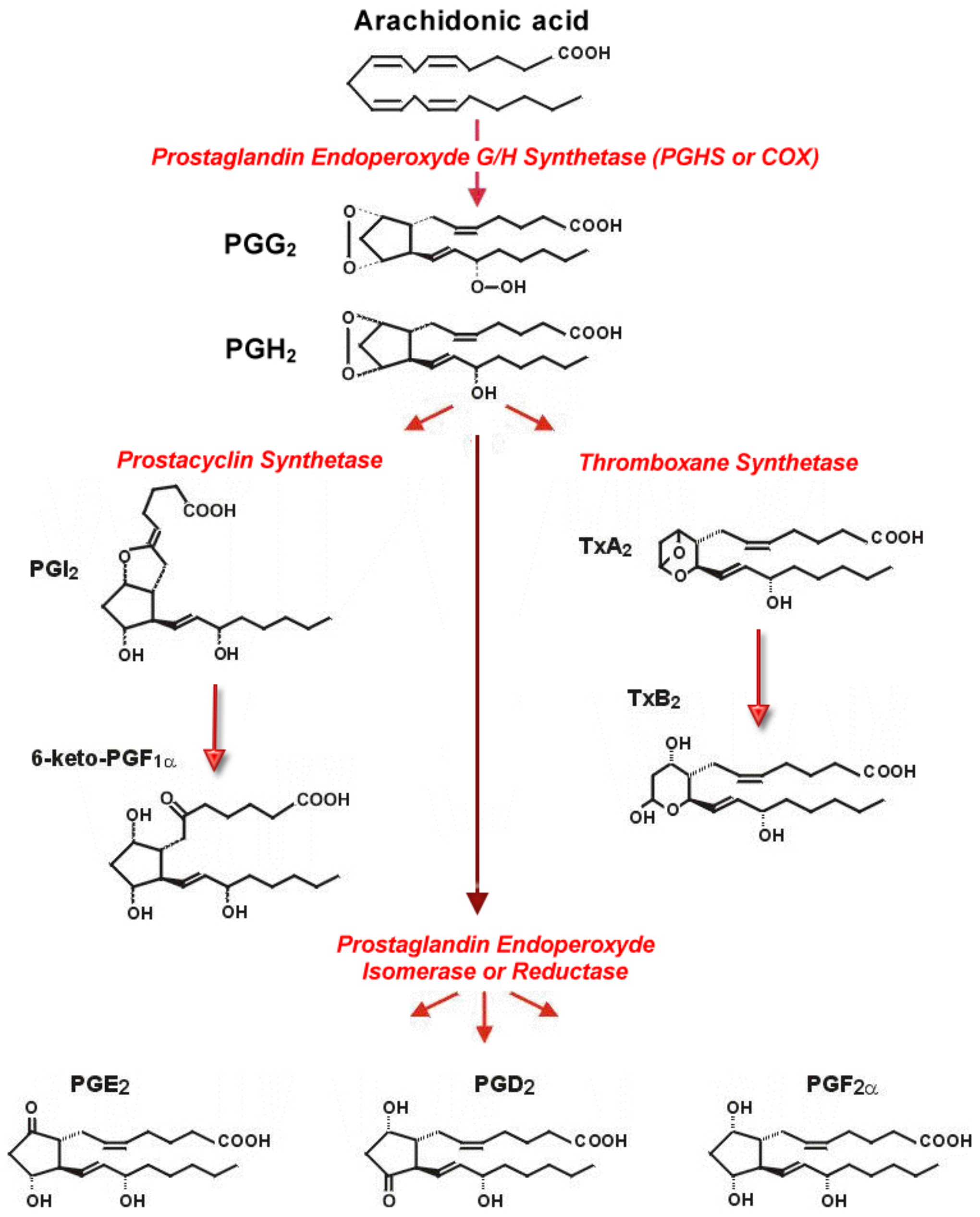

**FIGURE 1**. Principal prostanoids derived from arachidonic acid.

At the $EP_1$ receptor, initial work[15] demonstrated that various prostanoid analogs exhibited different potencies. The data have shown that the $EP_1$ receptor is sensitive to alterations at the C-15 position. An inversion of the stereochemistry at this position (15(R) configurations) induced substantial reductions in potency. In addition, methylation at the 15-position while maintaining the (S) configuration caused only minor reductions in potency. Therefore, the C-15 position was crucial for the potency of a variety of PGE analogues at the $EP_1$ receptor. Furthermore, oxidation of the hydroxyl to a ketone also resulted in reduced potency.

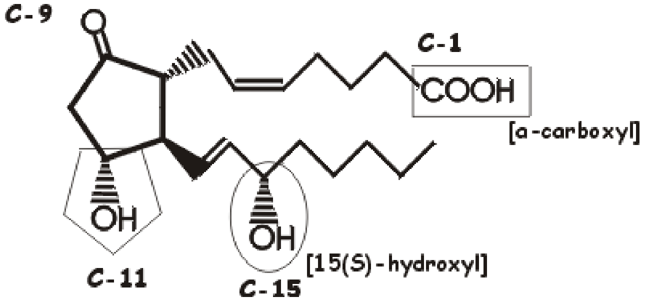

**FIGURE 2**. Structure of prostaglandin $E_2$.

Interestingly, when misoprostol free acid, a derivative of $PGE_1$ (racemic at the 16 position) was used, the potency was reduced 100-fold relative to $PGE_1$ and over 300-fold relative to $PGE_2$. In contrast, Abramovitz et al.[16] previously reported that in the human $EP_2$, $EP_3$, and $EP_4$ receptors in $[^3H]PGE_2$ radioligand competition binding experiments, misoprostol free acid was only slightly less effective than $PGE_2$ (7- to 30-fold). Together these observations suggested that the 15-hydroxyl, may not play a critical role in these latter EP receptors, whereas a marked effect was observed at the $hEP_1$ receptor. Thus, specific ligand alterations lead to distinct receptor activation.

Alterations in the carboxylic acid moiety at C-1 were also found to have significant deleterious effects on affinity and potency. In fact, the importance of the C-1 carboxylic acid moiety for binding to the $EP_2$[16,17], $EP_3$[18], and $EP_4$[16,17] receptor subtypes was reported to be essential for activation of all EP receptors. These observations suggested that there was a common characteristic found within the different EP receptor structures that involved the C-1 recognition region of the ligand.

In conjunction with these results, the observations derived from mutagenesis investigations[19,20] as well as the evidence from modeling studies[21] indicated that the C-1 carboxylic acid could interact with the arginine residue in the seventh transmembrane (TM) domain. This residue is present in all prostanoid receptors. These observations suggested an essential role for the seventh TM domain in prostanoid ligand binding. Since the carboxylic acid of prostanoids can interact by forming both ionic and hydrogen bonds, Chang et al.[22] initially suggested that the hydrogen bonds were sufficient for activation. However, sulprostone and enprostil retain potency, but have modified α-carboxyl groups. These observations led Ungrin et al.[15] to suggest that the retained potency was probably due to the presence of a phenoxy substitution at the C-16 position. Interestingly, earlier reports had suggested that acidity at the C-1 position was more important than steric bulk for activity[23]. This may also partially explain the potency of sulprostone at the human $EP_1$ receptor subtype.

In addition to these data, either removal of the hydroxyl group (C-11 position) or inversion of the stereoselectivity at the C-11-position also significantly reduced the potencies of a number of compounds. In fact, replacement of the 11-hydroxyl group with a ketone group significantly reduced the potencies of analogues, such as $PGE_1$, $PGD_2$, and $PGF_{1\alpha}$. These results suggested that the receptor at which the ligand acted was also capable of recognizing another moiety of the ligand.

Within the ω-tail region of the $PGE_2$, a number of substitutions caused reductions in potency, such as replacement with a cyclohexyl group at the C-18 position. However, most of these replacements led to only slight reductions in potency. Of considerable interest were the modifications resulting in a phenyl ring beginning at the C-18 position, which produced compounds exhibiting a marked positive effects on potency. Thus, 16- or 17-phenoxy alterations increased potency. These observations concerning phenylic changes improved potencies in structures that contained detrimental substitutions, for example, the 11-deoxy16,16-dimethyl-$PGE_2$ (potency increased approximately 25-fold) when compared with 11-deoxy-$PGE_2$. While these substitutions may offer a partial explanation for the potency of sulprostone and enprostil, they clearly suggested that, at least at the $EP_1$ receptor, there was a preference within the binding site. Therefore, ligand structural modifications clearly provided specific tools for selective activation of EP receptors.

Incidentally, chiral inversion at the C-8 position in $PGE_2$ also reduced potency. The compounds formed by this alteration are referred to as isoprostanes[24]. Although a study did suggest that isoprostanes may activate $EP_1$ receptors[25], the consensus today is that these agents exert their effects principally by activation of the TP receptor.

Other alterations in the $PGE_2$ molecule had only minor effects on potency; however, there were several exceptions, such as replacement of the oxygen at the C-9 position by a $CH_2$ group. This modification yielded a compound with an increase in potency of threefold, albeit no modification in affinity. In contrast, a substitution of 9β-chlorine dramatically improved the binding affinity with no apparent change in potency. This latter compound, now referred to as ZK110841, was reported to be a DP receptor agonist[1], however, ZK110841 is also known to exhibit high affinity not only for $EP_1$, but $EP_2$ as well as $EP_4$ subtypes[16]. Another interesting modification in the native ligand was achieved in a compound called butaprost. Butaprost free acid has been shown to be only twofold less potent than $PGE_2$, but the methyl ester was approximately 100 times less potent, suggesting that butaprost methyl ester was selective for $EP_2$ receptors over $EP_4$ receptor subtypes. In fact, recent data[26] demonstrated that butaprost free acid has lower intrinsic activity than $PGE_2$, suggesting that the compound is a partial agonist.

These chemical alterations in the ligand provided the initial basis for the development of both agonist and antagonists, since they have unraveled the principal and most sensitive regions of the native molecule, specifically for the agonist-EP receptor activity, namely, the hydroxyl group at the C-15 position. However, these investigations have provided data and tools well beyond the ligand-$EP_1$ receptor activation. The pharmacological profiling of the compounds derived from $PGE_2$, either by radioligand binding assays or through functional studies using recombinant systems, have been extended to include valuable tools, such as sulprostone, misoprostol, GX63799, AH23848B, and ONO compounds. While most prostanoid agonists do not have absolute selectivity, some discrimination between the receptors has been suggested.

The published data regarding $PGD_2$ synthetic agonists is less exhaustive than that reported for the modifications of native $PGE_2$. Initial studies were based principally on *in vitro* systems, either tissue contraction and/or relaxation[27,28] or neurotransmitter release assays[29], and against a limited number of prostanoid receptor subtypes. However, several investigations have now addressed this issue using recombinant receptor expression systems[30,31]. In competition assays, while the natural prostanoids exhibited comparatively low receptor-subtype selectivity, a series of synthetic DP receptor agonists, namely, SQ27986, ZK118182, and RS93520, were shown to exhibit a greater degree of selectivity for the DP receptor when compared with data derived from the $EP_3$, FP, IP, and TP receptors[32]. These investigators reported that SQ27986 exhibited the highest selectivity for the DP receptor. Interestingly, ZK110841, although exhibiting similar potency to ZK118182 for the DP receptor in $[^3H]PGD_2$ binding to DP receptors on platelet membranes, also exhibited high affinity for the human EP2 and EP4 receptor subtypes[31]. While SQ27986 had long been considered the reference molecule for activation of the DP receptor, other compounds have now emerged, notably the ONO agonists[33]. In addition, a $DP_2$ receptor agonist (15R-$PGD_2$) has been synthesized and the inversion of the configuration at C-15 of $PGD_2$ apparently results in enhanced $DP_2$ receptor activity with no effect on $DP_1$ receptor subtypes[34].

As has been indicated for $PGE_2$ (see above) structural modifications of $PGF_{2\alpha}$ have likewise been associated with FP receptor activation[35]. These investigators reported that the C-1 carboxylic group was essential for binding to the FP receptor, whereas chiral permutations of the hydroxyl group at the 15-position as well as substitutions at the ω-chain appeared to be of only minor importance for reductions in binding. Indeed, replacement of the carboxylic acid group by larger moieties, such as either acylsulfonamide or tetrazole, significantly decreased binding affinity. Previous work had shown that substitutions with bulkier anionic or neutral groups also markedly reduced binding. These observations led Schuster et al.[35] to suggest that the C-1 moiety interacts with the FP receptor in an ionic- and stereospecific manner. These observations strengthened the notion that the FP receptor may have a sterically restrictive pocket for the C-1 group of the ligand.

The majority of these investigations were inspired by an interest to establish prostaglandin agonists with selectivity for specific receptors so that the physiological significance of the receptors could be defined. These strategies also led to the development of specific antagonists. While this direction has generated a considerable amount of information for prostanoids, the approach to the other major metabolites of arachidonic acid, namely, the leukotrienes, was targeted principally for the development of antagonists. This was based on the early observations that the leukotrienes were associated with bronchoconstriction and cellular migration of inflammatory cells in the chronic respiratory disease asthma[36]. A compound that blocked the airway muscle constriction was thought, and eventually was shown, to possess therapeutic value in patients with this respiratory disorder. Therefore, the initial effort made to elucidate the structural alterations of the leukotrienes was undertaken with the essential purpose of developing antagonists.

The structures of the leukotrienes are presented in Fig. 3 and suggest that the leukotrienes exhibited structural moieties that were analogous in many ways to the entities found in the prostaglandin structures.

As previously mentioned, alterations in the prostaglandin structures were performed essentially to develop selective agonists, whereas the leukotrienes that had been associated with airways disease led to efforts to design leukotriene receptor antagonists. This undertaking was initially hindered by two major problems. First, the unstable triene-containing chain was difficult to replace and second, chemists were confronted with the problem of eliminating the cysteinyl-leukotriene (cysLT) agonist activity and transforming the molecule into a compound with antagonist activity. A number of approaches were used and efforts centered on replacing the unstable triene with a set of aryl-containing groups. The addition of a peptidyl chain, and deletion of one or more of the acid moieties, established compounds that had little or no agonist activities. Other structural modifications were based on the homo-cinnamyl triene with shortened links to the carboxylic acids. A third procedure was the incorporation of both hydroxyacetophenome and chromone carboxylic acid motifs that were derived from FPL55712 into a lipid backbone, since FPL55712 was the first compound to be described as an antagonist to SRS-A[37]. However, one of the most successful leukotriene antagonists was montelukast sodium[38]. This compound was based on a quinolone-containing structure that, to a limited extent, mimicked the olefin backbone of the cysLTs. Addition of a dithioacetal linkage was first incorporated and one of the carboxylic acid moieties was replaced by an amide. This compound exhibited significant potency and efficacy in oral administration. In addition, a resolution of the enantiomers gave rise to verlukast[39], a compound with considerably improved clinical effects over MK571[40]. Other approaches were also used to develop backup compounds, such as pyridine analogs of Singulair[41].

The message derived from these investigations was that the native leukotriene structure was used to develop compounds in which the agonist activities were abolished, thereby providing a therapeutic compound, that is, a drug that blocked the airway bronchoconstriction in asthmatics. Although a number of diverse compounds were developed, many shared identical structural elements with the agonists and, therefore, exhibited common binding sites for both the endogenous ligand and the antagonists. A number of attempts were made to construct a pharmacophoric model. Young[42,43] initially compared the molecular structure of $LTD_4$ and $CysLT_1$ antagonists. These studies concentrated on the essential requirements for the interaction between the leukotriene moieties and the receptor.

These investigators suggested four structural aspects (Fig. 4) for the leukotriene agonists/antagonists, namely, (1) a lipophilic anchor (side chain), (2) a central flat unit (triene), (3) an ionic pocket (peptide and/or carboxylic acid group), and (4) a hydrophilic site (acidic group). Since this description, other workers have further refined this conceptual model to a three-dimensional one[44,45,46,47]. Interestingly, the three-dimensional models have suggested that all $CysLT_1$ antagonists may not bind to the same site or in the same manner as the endogenous ligand. This had been suggested in a previously published report using guinea pig lung preparations[48]. In this latter study, the authors showed that the potency of ICI198615 decreased markedly in displacing bound $[^3H]$-$LTD_4$ following blockade of the arginine residues, whereas $LTD_4$ was unaffected. Therefore, further modeling may be necessary to characterize

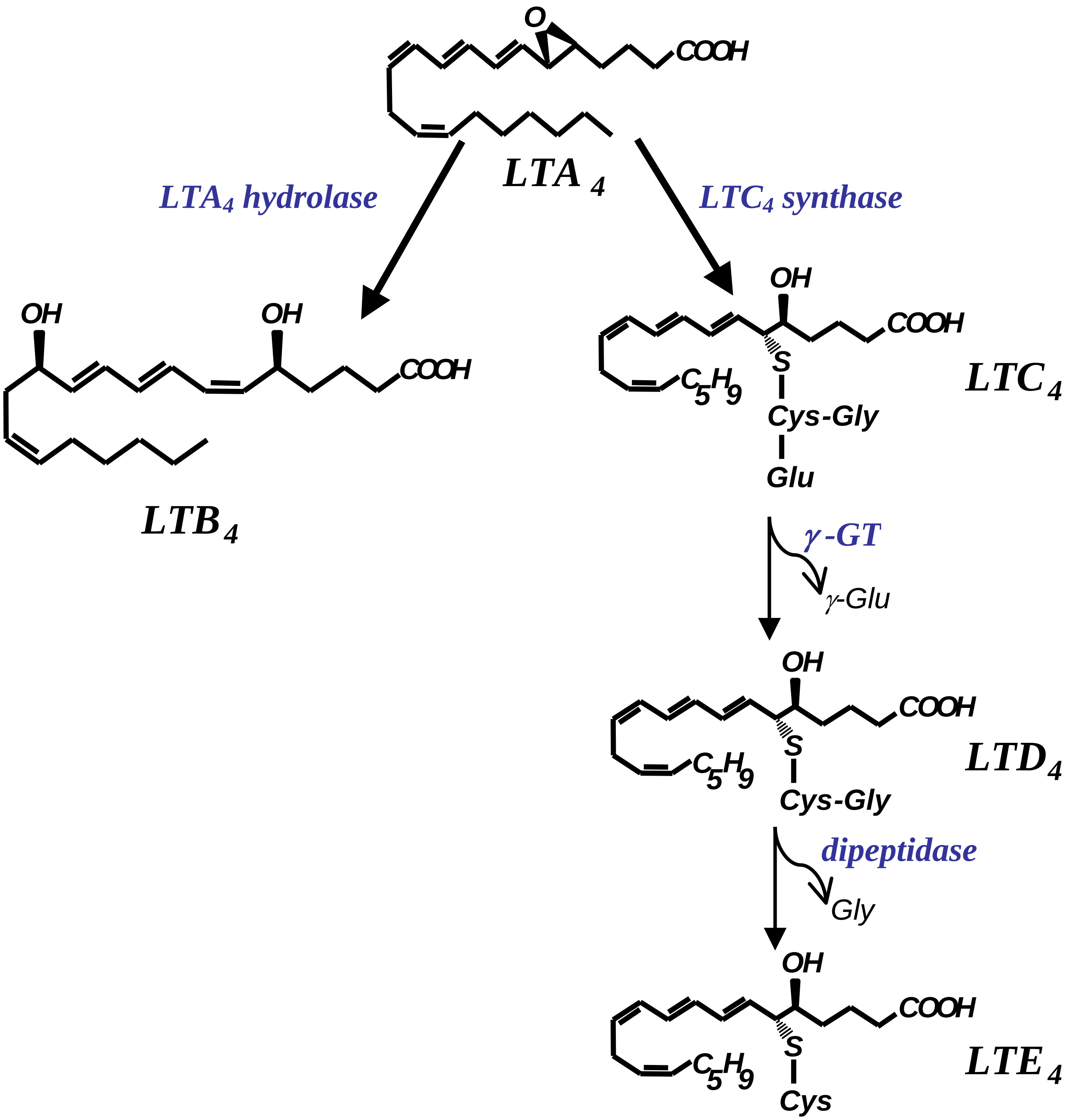

**FIGURE 3.** Schema of the leukotriene metabolic pathway.

how the $CysLT_1$ antagonists differ from the endogenous ligand interactions at the $CysLT_1$ receptor. In addition, certain $CysLT_1$ antagonists that contain flavones (structurally rigid compounds) may bind differently when compared with compounds containing quinolines (flexible compounds), therefore, a model of antagonist binding may have to include structural comparisons between different groups of antagonists.

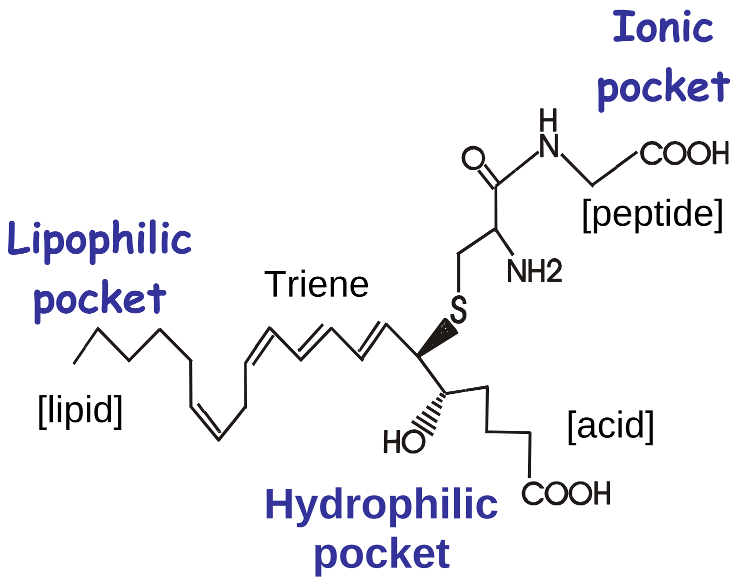

**FIGURE 4.** Structure of $LTD_4$.

## GENETIC ASPECTS OF EICOSANOID RECEPTOR STRUCTURES

The evolutionary relationships among the eicosanoid receptors have been described by the construction of phylogenic trees based on the DNA sequences for both the prostaglandin and leukotriene receptors. These analyses generally show that receptors that share coupling to the same second messenger are more closely associated than receptors that share the same native endogenous ligand[49,50]. This is particularly documented for the prostanoid receptors. For example, IP, DP, $EP_2$, and $EP_4$ all stimulate adenylate cyclase and lie in the same branch of the phylogenic tree, whereas $EP_1$, FP, and TP are coupled to phosphatidylinositol hydrolysis and lie in a separate branch. Of further interest is that members of this latter group are more closely related to each other than they are to $EP_3$, which is coupled to the inhibition of adenylate cyclase. The significance of the phylogenic tree analysis suggested two fundamental points. First, the second messengers and their coupling mechanisms must be considered as fundamental components in the receptor structure and second, the ancestral prostanoid receptor was probably an EP receptor from which other prostanoid receptors were subsequently derived.

Although distinct genes encode prostanoid receptors and are partially responsible for the heterogeneity of these receptors, variations have also arisen from alternative mRNA splicing. Of the eight prostanoid receptor subtypes, three of them have added to the receptor diversity by generating different isoforms due to mRNA splicing. Presently, the mechanisms involved in the control of the expression of these different isoforms remains to be established. In the case of the FP and $EP_3$ splice variants, these isoforms are thought to be associated with either truncated exons or with different combinations of exons. However, under some conditions, a specific mechanism for splice variants may exist, as in the case of the TP receptor[51]. These investigators showed that in the $TP_\beta$ receptor isoform, a 659-bp intron is spliced from the TP receptor pre-mRNA at an intron-exon boundary at a region in the gene that corresponds to a position approximately nine amino acids into the carboxyl terminus. In contrast, in the $TP_\alpha$ isoform, the 659-bp intron is not spliced out during the processing of the mRNA, so that intron that is spliced out in the generation of $TP_\beta$ is retained and becomes an exon. Thus, there is a failure to use a potential splice site. An explanation as to why this occurs remains to be established. Incidentally, this mechanism does not appear to be a common phenomenon for GPCRs, suggesting that prostaglandin receptors may be unique, albeit there is some evidence to suggest that rhodopsin and the dopamine receptors may also exhibit this phenomenon. In any event, what these observations suggest is that the regulation of prostanoid receptor number occurs not only at the level of the protein, but also at the level of the mRNA.

Interestingly, the human $EP_3$ gene is rather unique in the GPCR superfamily in that the nine $EP_3$ mRNAs are generated in multiple fashions by alternative mRNA splicing, and contribute to the generation of functionally different receptors from this gene. The human $EP_3$ receptor is known to have several spliced variants (eight functional), and there are two isoforms for both the human TP and the human FP receptor. In addition, the alternate splice variants for $EP_3$, TP, and FP are all identical throughout the seven transmembrane domains, but diverge approximately nine to 12 amino acids into the cytoplasm carboxyl terminus. One of the $EP_1$ isoforms has a truncated intracellular tail that does not couple to any signal transduction cascade. This $EP_1$ receptor isoform inhibits signaling at coexpressed $EP_2$ or $EP_4$ receptors, possibly by acting as a dominant-negative receptor as has been suggested by Okuda-Ashitaka et al.[52]. These observations suggest that the TM domain alone in the $EP_1$ truncated isoform may, in part, be responsible for the functional data and also interfere with signaling mediated by other prostanoid receptors.

## EICOSANOID RECEPTOR STRUCTURE

While the molecular weight of the eicosanoids is between 300 and 700, the receptor at which they act is approximately 100 times larger. This poses an interesting problem as to how a small molecule can fit and activate such a relativity huge membrane complex. Presently, the structural aspects of the eicosanoid receptor-ligand interactions have not been completely elucidated. However, considerable work has shown that there may be two components for ligand binding, one a critical amino acid and the second, neighboring amino acid residues, all of which are found principally in the seven TM domains. This is usually referred to as the “receptor binding pocket”. In addition, there may be accessory binding sites for ligands in which the interaction with the GPCR is less profound.

## RECEPTOR TRANSMEMBRANE (TM) DOMAINS

Hibert et al.[53], in a mutagenesis study of GPCRs for small ligands, suggested that the ligand binding pocket for these receptors was formed by the seven TM helices. Subsequent to these early observations, a conserved arginine in the seventh TM of the prostaglandin receptors had been identified as a critical determinant for prostaglandin receptor-ligand interactions[54]. This was also supported by the observation that a region in the seventh TM contains about 15 residues that are highly conserved for all these receptors. A point mutation in the seventh TM domain of the thromboxane receptor, namely, tryptophan at position 299, when substituted for a leucine residue allowed discrimination between agonist and antagonist binding sites[55]. Dorn et al.[56] later demonstrated that the first TM also played a role in the affinity ligand binding to the TP receptor. In addition, results obtained by Kedzie et al.[57] showed that a mutation (Leu-304 to Tyr) in the seventh TM domain of the $EP_2$ receptor caused a 100-fold increase in the affinity for iloprost. These data complemented the work previously published by Kobayashi et al.[58,59] who reported that the first, second, and seventh TM domains participated in the ligand binding of the DP and IP receptors. These latter investigators postulated that the first to third TM domains were involved in the recognition of the prostanoid ring structure, whereas the seventh TM domain was associated with the recognition of the side chain. However, recent information has suggested that these regions may not be the only ones implicated in the binding of the ligand to the receptor pocket (see below).

There is presently little information available concerning the role of the TM domains for the $CysLT_1$ and $CysLT_2$ receptors during receptor activation by the endogenous ligands. Most investigations concerning the leukotriene-receptor interactions have explored only the leukotriene $B_4$-BLT receptor interaction.

The initial report by Chiang et al.[60] suggested that exchanging major components in the $BLT_1$ receptor significantly modified the $LTB_4$ binding, albeit the C-terminus apparently had no affect. Further work[61] showed that the $LTB_4$ binding residues were found in the portion of the BLT receptor that was closer to the extracellular portion of this GPCR. Recently, in an excellent study by Sabirsh et al.[62], residues from the third and fifth TM helices were implicated in the binding pocket since the carboxylate group and the two hydroxyls of $LTB_4$ interacted with residues (Arg-178 and Glu- 185) in the fifth TM as well as with Val-105 and Ile-108 in the third TM. Of considerable interest were the observations that molecules lacking the C-12 hydroxyl bound poorly and failed to activate the $BLT_1$ receptor. These observations were corroborated with other structural modifications, such as adding a hydroxyl to the ligand at the ω-carbon 20, which also had little effect on ligand affinity. These results, as have been previously shown for the prostanoid ligands, suggested that relatively small alterations in ligand structure modify the stereoselective action of the ligands at the receptor. These investigators modeled their results and suggested that the $LTB_4$ binding pocket was situated between the third, fifth, sixth, and seventh TM helices. In this model, the specific cysteines did not appear to be involved in the binding, albeit necessary for the integrity of the receptor as had been reported by Banères et al.[63,64]. Incidentally, these latter investigators demonstrated that cysteine-serine mutations did play a role in the ligand binding, therefore, whether or not the cysteines are involved remains to be clarified.

Interestingly, the extracellular surface of the $BLT_1$ receptor has not been systematically explored in relation to ligand-receptor interactions. Further work will be necessary, therefore, in order to establish the role of the extracellular loops in relation to the binding pocket.

## RECEPTOR EXTERNAL LOOPS (EL)

While most of the amino acid conservation occurs in the TM helices, the N-terminal portion of the second extracellular loop (connecting the fourth TM domain with the fifth) also contains a cluster of seven residues within which there is a nonvariable triplet (Trp-Cys-Phe). Evidence for the role of the second EL in prostaglandin ligand-receptor binding was substantiated in an elegant series of mutagenesis studies on the $EP_3$ receptor[18] as well as on the $EP_2$ and $EP_4$ receptors[17]. These experiments revealed a crucial role for the amino acid threonine. In the case of the $EP_3$ receptor, a mutation of threonine 202 in the second loop produced proteins with increased ligand affinity. In addition, the hydroxyl moiety on the side chain of this conserved residue in the second loop of the $EP_2$ and $EP_4$ receptors was shown to be essential for ligand binding based on the data obtained from the threonine to serine and threonine to alanine mutations. Interestingly, among the prostanoid receptors, both the human TP and IP receptors contain a serine at the corresponding (analogous) conserved position as threonine, a residue that is found in all the other prostanoïd receptors. A replacement of the Cys-204 residue in the TP receptor with serine leads to a complete loss of binding and signaling[65]. Furthermore, within this highly conserved portion of the second extracellular loop, substitutions at the other conserved positions Gln-198, Trp-203, Cys-204, and Phe-205 did not affect ligand binding or signal transduction. These observations were the initial suggestions that determinants for prostanoïd receptor structure and/or function did not lie exclusively in the TMs[66]. These data were similar to that reported for the adenosine $A_2$ receptors containing single point mutations in glutamate residues in the second extracellular loop that were unable to bind radioligands. In addition, Olah et al.[67] demonstrated that substitutions of the distal 11 amino acids of the second EL in the adenosine $A_2$ receptor resulted in drastic changes in affinity for adenosine receptor agonists and antagonists. Both the prostanoid and adenosine receptors contain at least one cysteine residue within the region of the second EL, and these cysteine residues are thought to form disulfide bridges that bring the second loop in physical proximity to the ligand binding pocket. However, this was shown to be of little importance, since mutagenesis of the conserved Cys-204 in the second EL of the $EP_3$ receptor had no effect on receptor function or ligand binding[18].

While the picture regarding the structure of the prostanoid receptors is not complete, the above observations clearly indicate that the second EL must be considered seriously as a major component for ligand-receptor interactions. In fact, a considerable amount of evidence accrued based on work concerning the TP receptor. Initially, the report by Funk et al.[55] provided an impetus to investigate the functional consequences of substitution mutagenesis of various amino acid residues within the structural identity of the prostanoid receptors. As mentioned previously, the amino acid sequence in the seventh TM is conserved for all the known TP receptors and, therefore, may not be helpful for defining differences between the naturally occurring receptors.

An initial report by Dorn et al.[56] demonstrated a role of disulfide bonds in platelets, since dithiothreitol treatment decreased ligand binding capacity. These observations were soon followed by a report[65] that demonstrated specific mutations of serines, for cysteines in either the first (position 105) or the second (position 183) EL of the TP receptor led to markedly reduced binding and suggested the existence of a critical disulfide bond between these two positions. These observations were further supported by the results from other investigators in the same year[68]. In addition, this latter report also highlighted a distinct role for the cysteine at position 102 since this residue is not found in other prostanoid receptors. The data indicated that mutation of Cys-102 to a serine abolished [$^{3}$H]SQ29548 binding activity. Together, these findings indicated that the cysteinyl residues in the hydophilic first and second extracellular domains were involved in the ligand binding. In addition, these investigators also indicated a role for the serines (positions 201 and 255) in the fifth and seventh TM, since mutations of the serines to alanine altered [$^{125}$I]BOP without affecting the antagonist [$^{3}$H]SQ29548, suggesting that the hydroxyl group of the serines may interact with $TxA_2$ analogs possessing a 15(S)-hydroxyl group. These results also suggested that ligand binding affinity was principally determined by multiple contiguous or widely separated amino acid residues. Therefore, identification of the ligand pocket was thought not likely to be accomplished by a simple substitution mutagenesis of single amino acids.

To this end, a number of studies were undertaken utilizing regional exchanges between related receptors in association with either the increase or loss of ligand binding. Dorn et al.[56] explored different regions using TP chimeric receptors constructed from both human and rat. Their data demonstrated that multiple regions of TP receptors, including the first TM domain, were necessary for high-affinity [$^{125}$I]BOP binding. In fact, they reported that within the first TM, the combination of Leu-37 with either Ala-36 or Gly-40 was crucial for high-affinity binding. These latter observations led to the suggestion that these amino acid residues may act to control the space in the ligand pocket, since substitutions at these positions permitted the “loss” of high-affinity binding to be “rescued”. In addition, these investigators also indicated that a mutated human TP receptor, which lacked the intracellular carboxyl terminus, exhibited normal human TP receptor binding properties. Since there is considerable variation in the carboxyl tail between the hTP receptors ($TP_\alpha$ and $TP_\beta$), these observations suggest that ligand binding affinity is independent of intracellular carboxyl terminus and received further support from the work of Ruan et al.[69]. The latter investigators employed another strategy for exploring the ligand-receptor binding domain. In this study, the investigators took advantage of the observation that the IP receptor shared a similar primary structure in the extracellular loop with those of the TP receptor. Since 40% of the residues in the second EL between these receptors are identical, they constructed the peptide domains based on high homology scores by the homology modeling approach using the NMR structures of the TP EL peptide as templates and then configured the peptide to the seven TM template model (based on rhodopsin). While these investigators previously demonstrated that the antagonist binding recognition pocket was principally formed by the second EL, and the disulfide bond between the second EL and the first EL[70,71], the recent work[69] showed that a key residue (Arg-173) in the second EL domain was crucial for the ligand binding. In addition, Turek et al.[72] also reported that the third EL contains an important amino acid binding site. Together, these results provided sufficient support for the role of the ELs in the ligand-receptor binding interaction.

There are also a few reports suggesting that N-linked glycosylation is a pertinent determinant not only in the affinity, but also the specificity, of the $EP_3$ receptor for ligands[73]. Furthermore, conservation of the N-linked glycosylation sites at Asn-4 and Asn-16, located within the extracellular amino acid terminal region of this receptor, has also been suggested to play a role in ligand activation of the TP receptor[74].

The information available concerning the ELs of either the CysLT or BLT receptors is extremely limited. Presently, only the sequence of the ELs are known for the CysLT receptors, and most work on the structure of the BLT and CysLT receptors have explored only the C-terminus.

## RECEPTOR CYTOPLASMIC FACE

In addition to the observations concerning the extracellular loops, other structural aspects have been reported to play a role in the ligand-eicosanoid receptor interaction. As previously mentioned, cysteines had been suspected to modulate the ligand-GPCR activities. This was essentially thought to be due to their ability to form intramolecular disulfide bonds[56]. When the deduced amino acid sequence of the various prostanoid receptors was obtained, several cysteines were found to be present in the different structural components of these receptors. For example, in the human TP, cysteines are found in the extracellular amino terminus, the first and second ELs, as well as in the third intracellular loop (IC). Using chimeric receptors, D'Angelo et al.[65] demonstrated that an introduction of either a serine or alanine for the cysteine at Cys-223 in the third IC markedly altered calcium signaling, but had little effect on ligand binding properties. Furthermore, these investigators also showed that a change in the Thr-221 residue to Met also diminished cell signaling. However, modification of the cysteine found within the sixth TM (Cys-257) did not affect either ligand binding or calcium signaling. Interestingly, this cysteine residue is also found in IP, $EP_2$, and FP receptors, suggesting that this residue may play a similar role for each of these receptors. However, an analogous cysteine residue present in the $\beta_2$ receptor (Cys-285), when replaced, led to impaired cAMP accumulation[75]. These observations suggest that there are minor structural differences between these receptors (eicosanoid vs. $\beta_2$ receptor) even though both types are frequently modeled on the structure of the rhodopsin receptor. Together, these data indicated that the third IC was associated with cell signaling. However, receptor coupling to the G-proteins has also been noted for other IC loops. The first IC loop of the TP receptor was initially implicated in G-protein coupling by Hirata et al.[76], who reported a mutation of Arg-60 to leucine associated with a hemorrhagic disorder. Site-directed mutagenesis studies[65] also demonstrated that alterations (Arg-60 to leucine) led to a failure of the TP receptor to activate phospholipase C. Such observations support the notion that multiple components of the eicosanoid receptors are necessary for appropriate cytoplasmic protein coupling. In addition to these observations, where exchanges between different receptors were made between the bradykinin $B_2$ and the $EP_2$ receptors, these chimeric receptors have proved to be quite fruitful in determining the structural receptor-signaling cooperation[77]. While bradykinin activation of the $B_2$ receptor does not increase cAMP accumulation, $PGE_2$ activation of the $EP_2$ receptor is known to increase cAMP levels in cells significantly. An earlier publication[78] showed that wild-type Rat-1 cells do not express $EP_2$ receptors and that $PGE_2$ did not increase cAMP levels. Using this cell line, Pal-Ghosh et al.[77] performed a series of mutant constructs based on the second IC. Previous work also demonstrated that the $EP_2$ receptor contains six amino acids that appear to be a focal point for adenylate cyclase activation and insertion of this sequence alone into the $B_2$ receptor induced a marked elevation of cAMP production. These experiments were corroborated using other second IC exchange mutants in Rat-1 cells and all mutants showed a dramatic increase in the cAMP concentrations. The data supported the general notion that several distinct regions of the GPCRs, namely, IC3 as well as IC2, confer specific activity of receptors with their targeted G-protein.

Perhaps the most studied structural aspect of the eicosanoid receptors so far has been that of the carboxyl terminus (C-terminal region). The role of this region has been particularly well documented for several of the eicosanoid receptors. For example, removal of the C-terminal tail region in the TP[74], FP[79], $EP_4$[80], or IP[81] obliterates the cellular response, whether calcium mobilization, inositol phosphate generation, receptor phosphorylation, or receptor trafficking were monitored. However, other results have suggested that the initial studies involving short-term activation of receptors, for example, IP, may be quite different from results obtained after a more chronic treatment[82]. This has been described for the IP receptor, which may be sequestered in response to ligand activation via a protein kinase C (PKC)–independent pathway that is distinct from desensitization[83].

However, there are eicosanoid receptors that are quite different from other prostanoid receptors. This is particularly evident for the $CRTH_2$ receptor. The $CRTH_2$ receptor does not share homology with the DP receptor, but exhibits sequence similarity to the formyl peptide chemoattractant receptors (FPR[84]) and is also related to the eicosanoid-binding chemoattractant receptors, namely, BLT and ALX. Interestingly, the residues that have been reported to play a role in DP ligand binding are not conserved in the $CRTH_2$ receptor and data derived from studies with indomethacin and arylacetic acid NSAIDs[85] demonstrated differences in the binding of these agonist/antagonists at the DP and $CRTH_2$ receptors. These observations would suggest that $CRTH_2$ binds the prostaglandin ligand in a distinct fashion from DP receptors. Kobayashi et al.[59] reported that high-affinity binding in the DP receptor depended on the Lys-75 in the second TM domain. Further studies[86] demonstrated that a conserved region in the seventh TM domain, when modified, produced only slight alterations in $PGD_2$ binding. In contrast, replacement of Lys-209 in the fifth TM domain markedly reduced $PGD_2$ binding. These investigators also noted that specific residues (Thr-103 and Ser-114) in the peptide receptors (FPR and C5aR) played a crucial role in peptide binding, whereas the Ser-107 in the $CRTH_2$ residues did not. Furthermore, residues in the peptide receptors that are not involved in peptide ligand binding apparently play a role in $PGD_2$ binding in the $CRTH_2$ receptor. Generally, ligand binding for the DP and IP receptors involves the sixth and seventh TM domain, which recognizes the α-chain and terminal carboxylate of the prostaglandin ligand. In the TP and $EP_3$ receptors, an arginine in the seventh TM domain is associated with the carboxylate moiety. Hata et al.[86] suggested that the interactions of the $PGD_2$ carboxylate and the Lys-209 of the $CRTH_2$ receptor would orient $PGD_2$ in the receptor pocket in a different manner to that reported for the other prostanoid receptors. They also proposed for the $CRTH_2$ receptor that the cyclopentyl ring of $PGD_2$ lies between the third and fifth TM domain, which is quite different from the other prostanoid receptors where the ring structure is positioned near the first and second TM domains. Together, these observations and the models that are proposed suggest that there are both specific residues and residue regional clusters that permit the eicosanoid receptors to bind various ligands. In fact, with modern techniques for studying ligand-receptor interactions, the models are becoming more refined such that several specific amino acid clusters have been implicated for receptor activation. Receptor modeling now suggests at least four aspects associated with the ligand-receptor interaction, namely, a binding pocket, an accessory binding pocket, a lower TM domain movement, and distal TM stabilization[87]. Ligand binding to the receptor pocket induces a movement that provokes transient conformational changes in the receptor complex.

Conformational changes in GPCRs are intimately related to the exchange of GDP for GTP on the $G_\alpha$ subunit. This interaction leads to the dissociation of the α- and βγ-subunits. Subsequent to a G-protein activation, GPCRs are inactivated by a variety of mechanisms, namely, phosphorylation of cytoplasmic residues (Ser/Thr) by GRK (GPCR kinase), PKC, and cAMP-dependent protein kinase. This inactivation is associated with β-arrestin–dependent internalization of GPCRs[88]. Therefore, exposure of GPCRs to the endogenous ligand eventually leads to a low-affinity state that is known to be caused by the GDP-GTP exchange on the $G_\alpha$ subunit.

This receptor structure-G-protein interaction was nicely accessed using the $BLT_1$ receptor. To date, most investigations concerning the leukotriene receptors have dealt only with the structure-functional aspects of the C-terminus, specifically for the $BLT_1$ receptor. These reports have concentrated on the role of the eighth helix[89,90] in the $BLT_1$ structure. Together these pertinent structural observations led to the idea that the low- and high-affinity of the $BLT_1$ receptor state and the eighth helix were intimately linked.

The eighth cytoplasmic helix was originally described from the atomic structure of rhodopsin. This helix projects at a right angle from the C-terminus of the seventh TM and is anchored by palmitoyl residues to the cytoplasmic membrane surface. Okuno et al.[89] performed a series of experiments that demonstrated that the eighth helix of the $BLT_1$ receptor was involved in the detection of signals from the GTP form of the $G_\alpha$ subunit, and was associated with a conformational alteration of the receptor to release the ligand and stop the intracellular signaling. This suggested that conformational changes at this part of the receptor structure were related to receptor desensitization. In an initial elegant study, Gaudreau et al.[90] reported evidence that showed that a specific dileucine moiety in the eighth helix was intimately associated with receptor internalization. They hypothesized that the Thr-308 near the eighth helix may be targeted by GRK6 in the desensitization of $BLT_1$ receptors, following exposure to $LTB_4$[91]. In addition, they also suggested that phosphorylation of Thr-308 by GRK might simply unhook the C-tail structure from the membrane, thereby facilitating a conformational change to a Leu-304-Leu-305 moiety (dileucine formation). These observations stressed the notion that the eighth helix, when attached to the cytoplasmic membrane, was simply hiding the high-affinity sites, making them inaccessible to the G-protein. Thus, the eighth helix was detecting the status of the coupling of the $G_\alpha$ subunit as being either GTP bound or being anchored in the cytoplasmic membrane[92]. When the eighth helix was held at right angles to the seventh TM by the aromatic residues, the $BLT_1$ receptor was stabilized to a low-affinity state, that is, the $G_\alpha$ subunits were bound to GDP.

Recently, Basu et al.[93] have explored the $LTB_4$ binding site in $BLT_1$ using both computational modeling and site-directed mutagenesis in order to map the $LTB_4$-$BLT_1$ interactions. These investigators have elegantly demonstrated that the ligand receptor binding was intimately linked with the polar residues of the third, fifth, and sixth TMs and EL2, whereas polar residues in the second, third, and seventh TMs played a central role in the ligand-induced activation. These data provide an initial delineation of the ligand-induced receptor conformational changes associated with an activation mechanism.

There are also considerable data that suggest that receptors exist as homo- or heterodimers, and such conformations for the $BLT_1$ receptor have been documented. Of considerable interest was the initial observation that the dimerized $BLT_1$ receptor bound only one G-protein trimer to form a pentameric complex[63,64]. In addition, there are recent data to suggest that the $BLT_1$ receptor dimer with a single ligand-occupied subunit triggered G-protein activation. These data have contributed to the notion that the interaction of the G-protein with the dimer induced conformational constraints that prevented a symmetric functioning of this dimer[94]. However, there is a need to explore these changes further and to establish the role of dimerization in this class of receptors. In light of all these observations, the known sites of ligand-receptor prostanoid interactions are summarized in Table 1.

## RECEPTOR SIGNALS

While the focus of attention on the eicosanoid receptor structures is just beginning to be unraveled, a considerable amount of information is available concerning the cellular signaling cascades that are generated by the ligand-eicosanoid receptor interactions. Hata and Breyer[95] recently provided an excellent and thorough review of this area; only a few examples of the ligand-receptor structure-signals will be highlighted to demonstrate what has been suggested for the role of the C-terminus.

Since Slipetz et al.[80] clearly demonstrated that the C-terminus contained all the determinants that controlled agonist-induced $EP_4$ desensitization, sequestration, and phosphorylation, a further appreciation of the role of the C-terminus was assessed by examining the profiles of the G-protein coupling for the two sliced variants of the FP receptor.

The C-terminal tail of the $FP_\alpha$, but not the truncated $FP_\beta$, is phosphorylated by PKC, which leads to desensitization and internalization[109]. In contrast, $FP_\beta$ does not undergo ligand-induced internalization, but rather constitutive internalization[110]. This latter mechanism has been associated with $PGF_{2\alpha}$ activation of the Tcf/β-catenin cascade via a PI 3-kinase linked to the unstimulated β-catenin in the absence of the ligand. On ligand activation, the β-catenin complex is released, thereby increasing the levels of β-catenin and, subsequently, Tcf transcriptional activation[111]. Unfortunately the role of these two signaling pathways has not been fully investigated. Along similar lines, initial studies have reported that $TP_\beta$, but not $TP_\alpha$, undergoes ligand-induced internalization in a number of cell types[96]. These investigators went on to provide data demonstrating that the $TP_\beta$ also undergoes tonic internalization due to a specific moiety found in the proximal portion of the C-terminus[97]. Together, these observations suggested distinct signals for tonic- and agonist-induced internalization for these receptor isoforms, effects regulated by the C-terminus.

**TABLE 1**
**Prostanoid Receptors and Known Structurally Related Binding and Signaling Regions**

| Receptors | Ligand Binding Regions | Receptor Signaling Regions | Ref. |
|---|---|---|---|
| TP | First, second, third, fourth, fifth, sixth, and seventh TMs; first, second, and third ELs; N-glycosylation (amino acid terminal) | IC1, IC2, IC3, and C-terminus | [21,55,56,66,68, 69,70,71,72,76, 96,97,98] |
| $EP_1$ | | | [19,52] |
| $EP_2$ | First, second, sixth, and seventh TMs; second EL | IC2 and IC3 | [17,22,57,77,78, 99] |
| $EP_3$ | Fifth, sixth, and seventh TMs; second EL; N-glycosylation (amino acid terminal) | IC3 and C-terminus | [18,20,54,73,100, 101,102,103, 104] |
| $EP_4$ | Second EL | IC3 and C-terminus | [17,99,104,105, 106] |
| IP | First, second, third, sixth, and seventh TMs; first and second ELs | C-terminus | [58,59,81,82,83, 87,107,108] |
| DP | First, second, fifth, sixth, and seventh TMs; first EL | | [58,59] |
| $CRTH_2$ | Third and fifth TMs | | [84,85,86] |
| FP | | C-terminus | [35,79,109,110, 111] |

*Note:* There may be some differences based on cell types and species. Empty space indicates not explored. See text for other references.

The IP receptor also undergoes agonist-induced internalization and sequestration, which is independent from desensitization via a PKC-, GRK-, and arrestin-independent mechanism[81]. However, isoprenylation of the C-terminus is necessary for the agonist-induced internalization[107]. While isoprenylation was not necessary for binding, this was absolutely required for IP receptor activation of adenylyl cyclase via $G_{\alpha s}$ coupling and for efficient coupling to $G_q$/PLC as well as receptor internalization[107,108]. In fact, these latter investigators suggested that isoprenylation may be unique for the IP receptor.

In the case of the $EP_3$ receptor, where a considerable variation in the C-terminus has been observed, there are some interesting features to highlight. The $EP_3$ was initially thought to be coupled to the $G_i$-protein linked to cAMP inhibition[112], however, several splice variants were also coupled to $IP_3$ production[100,101]. Further evidence has been provided to demonstrate that truncation of the $EP_3$ to only 10 amino acids in the C-terminus led to a receptor that was incapable of activating $G_i$-protein[102], but was constitutively active. These observations indicated that the C-terminal domain was responsible for keeping the $G_i$-protein inactive as long as the receptor had not bound the ligand. These and other observations led to the notion that the C-terminal contained negative regulatory elements and, therefore, maintained a nonsignaling state for the ligand-free receptor[103].

Another interesting diversity in signaling, which arises in the C-terminus tail, has been noted in the $EP_2$ vs. $EP_4$ receptors. The former has a markedly shorter tail than the latter and this may explain, in part, why the $EP_4$ receptor undergoes a rapid desensitization as compared with that of the $EP_2$ receptor[99,105]. In addition, this difference in tail length has also been reported to be responsible for $EP_4$-induced internalization, whereas this was not observed for the $EP_2$ receptor when activated by the ligand[106].

While these data implicate the C-terminus as being critical for determining receptor G-protein coupling, this structural part of the receptor may not be the only determinant for the signaling interaction. Neuschafer-Rube et al.[104], using chimeric $EP_3$/$EP_4$ receptors in which the carboxylic terminus of the

$EP_3$ was replaced with that of the $EP_4$ receptor, demonstrated a similar potency to the wild-type $EP_3$ receptor. However, both receptors inhibited forskolin-stimulated cAMP. Since the wild-type $EP_4$ receptor had been associated with cAMP-increased production, the $EP_3$/$EP_4$ chimera should have exhibited a similar response. Therefore, these observations suggested that perhaps the IC3 may also participate in this G-protein phenomenon.

These examples indicate the diversity found in the C-terminus, which may explain the differences in agonist-induced trafficking linked to desensitization, internalization, and/or degradation of the receptors.

## CONCLUSION

To date, most attempts to infer the structure of the eicosanoid receptors have been based on indirect evidence and the rhodopsin template model. There are a number of highly conserved residues within the TM domains for the Group A receptors. However, the domains often differ with regard to the positions of the residues and, in some cases, even the absence or presence of specific residues in certain GPCRs, for example, proline. One basic message is that a residue positional diversity could account for the differences in ligand binding among the various GPCRs. Although the overall architecture of the rhodopsin template for the GPCRs remains quite valid, the side chains of the TM residues are highly specialized to interact with the chemical nature of the ligand and this local membrane environment reflects the mode of ligand access for receptor activation. However, until the structures are resolved and the ligand contacts identified, our understanding of the ligand-eicsanoid receptor interaction remains at best only suggestive.

There are several interesting observations concerning the structural aspects of the eicosanoid receptors that need to be summarized.

- The native endogenous ligand potency can be significantly altered by mutations of a single amino acid residue in the receptor structure.
- Nuances in the residues and their positions in the helices of prostanoid receptors exist when compared with those of rhodopsin.
- Agonists and antagonists may bind to structurally different entities of the eicosanoid receptors[98].
- While activation of the GPCRs results in an increase in mobility of the TM helices, there is no information concerning this aspect for the eicosanoid receptors.
- Eicosanoid receptor signals are intimately limited to the cytoplasmic face of the receptor principally involving the C-terminus.

In addition to these points, there are also some observations that need to be further explored. While the same ligand may activate different eicosanoid receptors, the ALX receptor has exhibited an unusual ligand diversity. Peptides have been reported to activate the ALX receptor and this binding is lost when the receptor is deglycosylated, whereas the $LXA_4$ binding is unaffected by such a treatment[113]. Under these conditions, one may speculate that the binding of the peptides is to a “weak or accessory site” of the receptor that may be principally located at the level of the extracellular residues of the ALX receptor[60]. Furthermore, $LXA_4$ activates the Ah nuclear receptor[114], an effect that has just recently become a subject of interest[115].

Evidence has also demonstrated that the cysLTs can activate the $P2Y_{12}$ receptor[116] and $cysLT_1$ antagonists block nucleotide activation of the $P2Y_{12}$ receptors[117,118]. Recently, both uracil nucleotides and cysLTs have been reported to activate the orphan receptor GPR17[119]. These observations suggest that the binding pocket(s) in different receptors have structural components that are closely related and may recognize a variety of ligands. However, the residues involved have yet to be demonstrated. In addition, these observations may also suggest that there is an interaction at a common G-protein signaling pathway.

There remain some classical pharmacological and binding studies that suggest the existence of other leukotriene receptors that have not been cloned[120,121,122,123]. These observations indicate that while the ligands are known, the sequence and structure at which they act have not been completely established.
